# Identifying and validating molecular subtypes of biliary atresia using multiple high-throughput data integration analysis

**DOI:** 10.3389/fimmu.2022.1008246

**Published:** 2023-01-12

**Authors:** Dingding Wang, Shen Yang, Yong Zhao, Yanan Zhang, Kaiyun Hua, Yichao Gu, Shuangshuang Li, Junmin Liao, Ting Yang, Jiawei Zhao, Jinshi Huang

**Affiliations:** Department of Neonatal Surgery, Beijing Children’s Hospital, Capital Medical University, Beijing, China

**Keywords:** biliary atresia, molecular subtypes, biliary fibrosis, immune, prognosis

## Abstract

**Background:**

Biliary atresia (BA) is the most common form of severe neonatal obstructive jaundice. The etiology and pathogenesis of BA are multifactorial, and different factors may interact to produce heterogeneous pathological features and clinical outcomes. Despite different pathological features, all patients received the same treatment strategy. This study performed integrative clustering analysis based on multiple high-throughput datasets to identify the molecular subtypes of BA and provide a new treatment strategy for personalized treatment of the different subtypes of BA.

**Methods:**

The RNA sequence dataset GSE122340 in the Gene Expression Omnibus (GEO) database was downloaded; 31 BA and 20 control normal liver tissues were collected at our center for transcriptome sequencing, and clinical and follow-up data of BA patients were available. Molecular subtypes were identified using integrated unsupervised cluster analysis involving gene expression, biliary fibrosis, and immune enrichment scores based on the transcriptome dataset, and the results were validated using independent datasets.

**Results:**

Based on the results of the integrated unsupervised clustering analysis, four molecular subtypes were identified: autoimmune, inflammatory, virus infection-related, and oxidative stress. The autoimmune subtype with a moderate prognosis was dominated by autoimmune responses and morphogenesis, such as the Fc-gamma receptor and Wnt signaling pathway. The biological process of the inflammatory subtype was mainly the inflammatory response, with the best prognosis, youngest age at surgery, and lowest liver stiffness. The virus infection-related subtype had the worst prognosis and was enriched for a variety of biological processes such as viral infection, immunity, anatomical morphogenesis, and epithelial mesenchymal transition. The oxidative stress subtype was characterized by the activation of oxidative stress and various metabolic pathways and had a poor prognosis. The above results were verified independently in the validation sets.

**Conclusions:**

This study identified four molecular subtypes of BA with distinct prognosis and biological processes. According to the pathological characteristics of the different subtypes, individualized perioperative and preoperative treatment may be a new strategy to improve the prognosis of BA.

## Introduction

Biliary atresia (BA) is the most common neonatal cholangiopathy and characterized by obstruction of the extrahepatic bile ducts and progressive liver fibrosis (1). The main treatment is Kasai Hepatoportoenterostomy (HPE), which may relieve biliary obstruction and restore bile drainage in some children but does not prevent progressive fibrosis (2, 3). Even if HPE was performed within 45 days after birth, the natural liver survival rate (NLS) was only 40.5% before the age of 15 years, and more than 75% of the patients still need to undergo liver transplantation (4). Due to the lack of knowledge of the etiology and pathogenesis of BA, all patients underwent similar surgery and adjuvant medical treatments. However, the disease progression and clinical outcomes of different patients showed significant heterogeneity.

The etiology and pathogenesis of BA are multifactorial, involving defective embryogenesis, genetic abnormalities, environmental toxins, viral infection, abnormal inflammation and autoimmunity, and susceptibility factors (5). Viruses and environmental toxins have been shown to injure cholangiocytes and activate innate and adaptive immune systems in BA animal models (6). These different factors could interact to produce clinical and pathological heterogeneity, which might lead to a distinct prognosis and require individualized treatment. However, the extent to which immune and molecular circuits are related to the pathological progression and clinical prognosis of BA in individual patients is not well established.

Therefore, in the current study, an integrated analysis involving gene expression, biliary fibrosis, and immunity was performed based on multiple BA high-throughput datasets, and the results were validated using independent datasets. Combining prognosis, biliary fibrosis, and immune and function enrichment scores, we identified four BA molecular subtypes with different prognosis and molecular characterizations. Immune, biliary fibrosis, and gene expression features that characterize each subtype indicate different pathological mechanisms. The results of this study may deep our understanding of BA and provide strategies for personalized treatment of different subtypes to release disease progression.

## Materials and methods

### Patients

BA liver samples were obtained from 31 patients who underwent HPE for type III BA at Beijing Children’s Hospital Affiliated to Capital Medical University from November 2017 to July 2021. The adjacent normal liver tissues of 20 children who underwent surgery for hepatoblastoma at our center during the same period were selected as controls. After the tissue was isolated, it was immediately placed in RNAlater and stored at -80°C. The diagnosis of BA is based on abnormal intraoperative cholangiography findings and histological manifestations of extrahepatic bile duct obstruction. All patients were routinely treated with steroids for 6 weeks after HPE. The degree of liver fibrosis was assessed by the Ishak fibrosis score of postoperative liver histological specimens, which was completed by two pathologists. Clinicopathological data were collected retrospectively. Postoperative cholangitis was defined as fever (>38.0°C) accompanied by elevated serum bilirubin (>2.5 mg/dL), leukocytosis with left shift and normal to acholic stools (7). Jaundice clearance was defined as a decrease

in total bilirubin levels below 1.5 mg/dL. Follow-up was performed 1, 3, 6, and 12 months postoperatively. The research protocol was approved by the Ethics Committee of Beijing Children’s Hospital affiliated with Capital Medical University (2019-k-386), and the parents of each patient signed an informed consent form.

### Liver stiffness measurement

Liver stiffness measurement (LSM) was performed with an Aixplorer ultrasound system (SuperSonic Imagine SA, Aix-en-Provence, France) and a SuperLinear SL15-4 probe. LSM was targeted at liver parenchyma free of large vessels, with the upper edge 0.5 to 1 cm away from the liver capsule. The region of interest for LSM was 1.0 cm diameter. LSM detection and analysis were performed by 2 sonographers.

### RNA extraction and sequencing

Total RNA was extracted from liver tissue. RNA libraries were constructed using a poly(A) protocol. cDNA libraries were constructed using a random hexamer primer, M-MuLV Reverse Transcriptase (RNase H Minus), DNA polymerase I, and RNase H. The Illumina NovaSeq 6000 system was used to sequence the cDNA libraries with paired-end 150 bp reads. The paired-end reads were gene-annotated by STAR 2.7.9a and quantified gene expression using Stringtie v2.1.4 with Ensembl gene annotation (GRCh38/hg38 assembly) (8, 9). Count data were normalized and processed using the DESeq2 package (10).

### Gene expression omnibus dataset collection and preparation

RNA-Seq datasets [GSE122340] were downloaded from the GEO database. GSE122340 included 171 BA liver tissue samples and 7 normal control samples. According to NLS, patients were divided into high or low NLS groups. The detailed grouping method was described in the original text (11). Based on batches, the 121 BA and 7 normal control samples were used as a training dataset, whereas the other 50 BA liver tissue samples were used for validation.

### Biliary fibrosis and immune cells index

The enrichment scores of activated hepatic stellate cells (aHSCs), activated portal fibroblasts (aPFs), and cholangiocytes were evaluated by single-sample gene set enrichment analysis (ssGSEA) in the “GSVA” R package (DOI:10.18129/B9.bioc.GSVA, version 1.42.0) to assess the degree of biliary fibrosis. The marker gene signatures of aHSCs, aPFs, and cholangiocytes were obtained from previous studies, respectively (12–14). The “xCell” (DOI:10.1007/978-1-0716-0327-7_19, version 1.1), a R package based on ssGSEA, was used to estimate the abundance scores of immune cells. Using the above method, we obtained an expression matrix of BA samples for subsequent analysis, which involved enrichment scores for biliary fibrosis and immune cells.

### Identification of molecular subtypes

Similarity Network Fusion and Consensus clustering (SNF-CC) algorithm in “CancerSubtypes” R package (DOI:10.18129/B9.bioc.CancerSubtypes, version 1.20.0) was performed to identify molecular classification of patients with BA in the training and validation datasets (15). Expression profiling of the top 5000 most variable genes, biliary fibrosis index, and immune cell index were selected for clustering. The silhouette width index was used to evaluate the reliability and accuracy of clustering results.

### Gene set variation analysis

The gene sets of c5.go.v7.4.symbols.gmt, and c2.cp.kegg.v7.4. symbols.gmt were downloaded from the Molecular Signatures Database to assess the molecular pathways and mechanisms. Using gene expression profiles, an enrichment score was calculated for each sample in each gene set using the previous method in the “GSVA” R package (DOI:10.18129/B9.bioc.GSVA, version 1.42.0) (16). The minimum gene set was set to 5, and the maximum gene set to 5000. Finally, the enrichment score matrix for each dataset was obtained.

### Weighted gene co-expression network analysis

The median absolute deviation (MAD) of each gene was calculated, and the top 10% of genes with the smallest MAD were removed. The goodSamplesGenes method of the “WGCNA” R package (version 1.70-3) was used to remove outlier genes and samples and build a scale-free co-expression network (17). Pearson’s correlation matrices and the average linkage method were both used for all pairwise genes. Then, a weighted adjacency matrix was constructed using the power function A mn=|C mn|^β (C mn = Pearson’s correlation between Gene m and Gene n; A mn= adjacency between Gene m and Gene n). To classify genes with similar expression profiles into gene modules, average linkage hierarchical clustering was conducted according to a topological overlap matrix (TOM)-based dissimilarity measure, with a minimum size of 30 for the gene dendrogram. We then merged modules with a distance of less than 0.25, and finally obtained 23 co-expression gene modules. Notably, genes that could not be assigned to any module were assigned to the gray module.

### Functional enrichment analysis

The core genes of each gene module were used for gene ontology (GO) and Kyoto Encyclopedia of Genes and Genomes (KEGG) analyses. The R package “org.Hs.eg.db” (DOI:10.18129/B9.bioc.org.Hs.eg.db, version 3.15.0) was used for gene annotations. GO and KEGG functional analyses were performed using the R package “clusterProfiler” (version 3.14.3). Differences were considered statistically significant at an adjusted p-value of less than 0.05.

### Protein-protein interaction network analysis

First, the hubgenes of each molecular subtype were obtained according to the WGCNA results. These hubgenes were imported into STRING (version 11.5) to obtain protein interactions (18). The visualized Protein-Protein Interaction (PPI) networks were subsequently obtained by the plugin Molecular Complex Detection (MCODE) of Cytoscape (version 3.7.2) (19, 20). Different colors in the PPI network represent each MCODE clustering module.

### Integrative classifiers for molecular subtypes

Significance analysis of microarray was used to obtain significantly different genes in each subtype. The R package “glmnet” (version 4.1-4) was used for dimensionality reduction and screening of clinical factors and signature molecules. Linear combinations of integrated clinical and molecular properties weighted by regression coefficients were used for the diagnosis of subtypes. The receiver operating characteristic curve (ROC) was used to examine the diagnostic efficacy of the classifier by the R package “pROC” (version 1.18.0).

### Statistical analysis

All data analyses were performed using R software (x64, version 4.1.3). Fisher’s exact test was used to compare categorical variables. One-way ANOVA or Kruskal-Wallis tests were performed to compare continuous variables between the different groups. The R package “pwr” (version 1.3 -0) was used to perform a power analysis using Cramer’s V (21) and Cohen’s f (22) as effect size. A two-sided *p* < 0.05 was considered statistically significant.

## Results

### Subtype identification of BA

**Figure 1:**
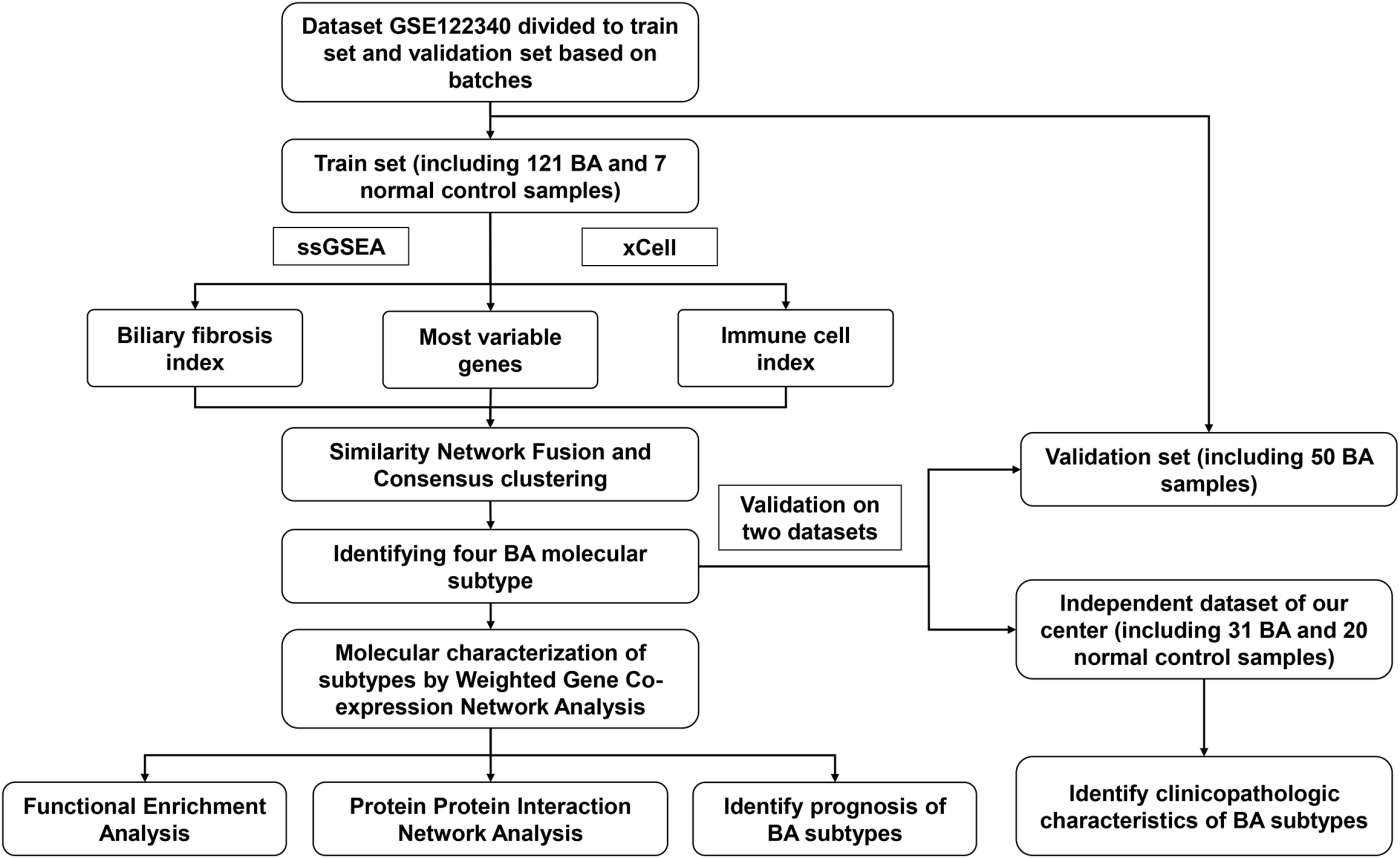
The flowchart of the integration analysis.

**Figure 2:**
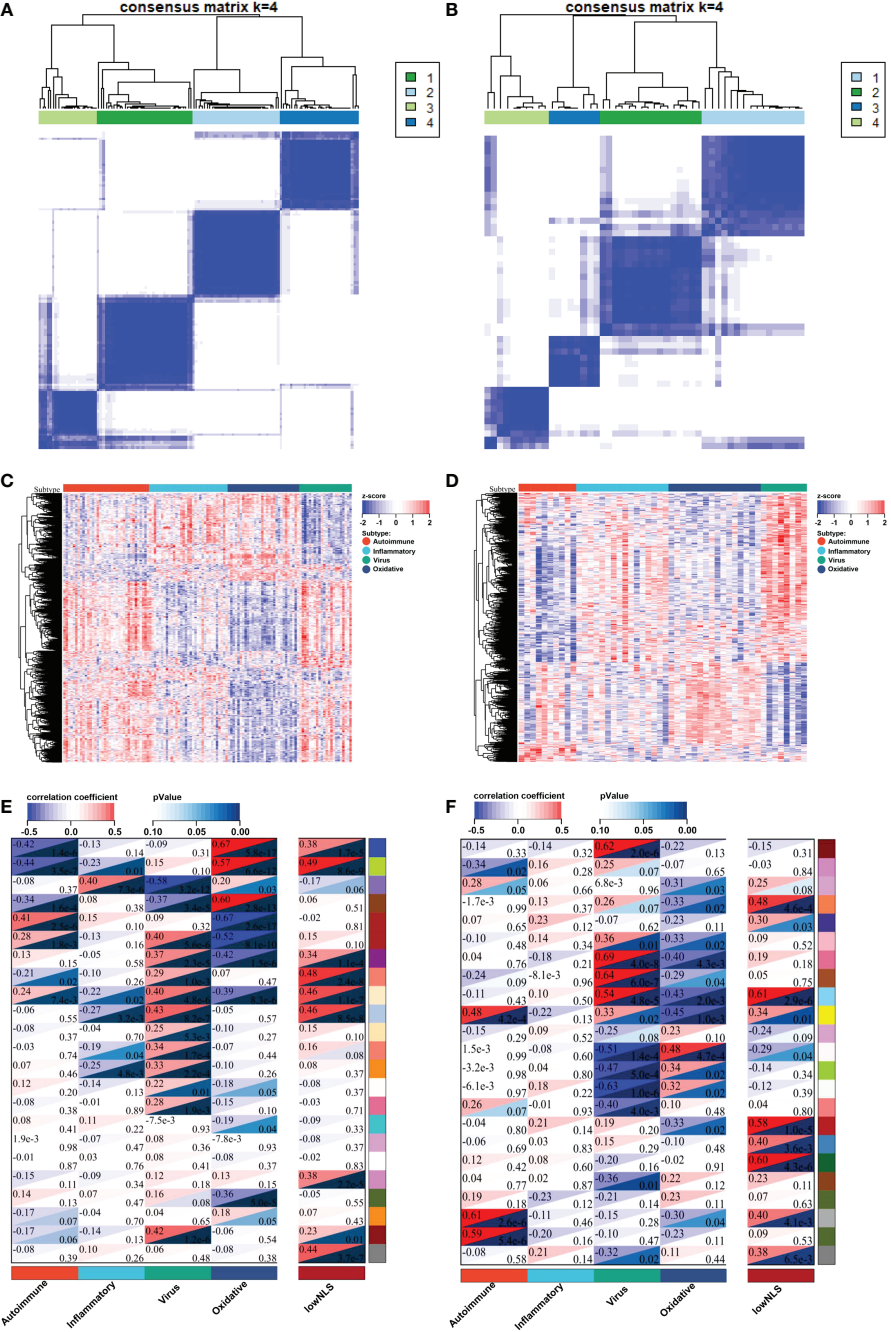
Identify four molecular subtypes of patients with BA in the training and validation datasets. By applying SNF-CC, training set **(A)** and validation set **(B)** were clustered into four distinct subtypes. GSVA similarly classified the samples into four subtypes in training set **(C)** and validation set **(D)**. Each subtype in training set **(E)** and validation set **(F)** was involved with different gene modules of WGCNA. BA, biliary atresia; NLS, natural liver survival; SNF-CC, Similarity Network Fusion and Consensus clustering; GSVA, Gene set variation analysis; WGCNA, Weighted Gene Co-expression Network Analysis.

The flowchart of the integration analysis was shown in **Figure 1**. In the training dataset of 121 BA liver samples, the relative enrichment of biliary fibrosis and immune cells was assessed using GSVA and xCell packages. We found significant differences between the BA and normal liver samples in fibrosis and immune cell types (**Supplementary Figure 1B**). By applying SNF-CC, the training dataset was clustered into four different subtypes. The silhouette width was 0.85, suggesting that the clustering results were reliable with high degree of cohesion and separation (**Figure 2A** and **Supplementary Figure 1A**). GSVA was then applied to detect differences in biological pathways among the four subtypes, which similarly divided the dataset into four subtypes (**Figure 2C**). In addition, WGCNA identified a series of gene modules representing different biological processes, and each subtype was associated with different gene modules (**Figure 2E**).

The validation was performed using a valid dataset. The 50 samples were similarly clustered into four distinct subtypes based on gene expression, immune, and biliary fibrosis, with a silhouette width of 0.77 (**Figure 2B** and **Supplementary Figures 2A, B**). Again, the GSVA results classified the samples into four subtypes and revealed distinct molecular pathways (**Figure 2D**). WGCNA also showed that each subtype could be distinguished using different gene modules (**Figure 2F**). These results above validated the reliability and reproducibility of the molecular classification.

### Gene functional annotation of molecular subtype

**Figure 3:**
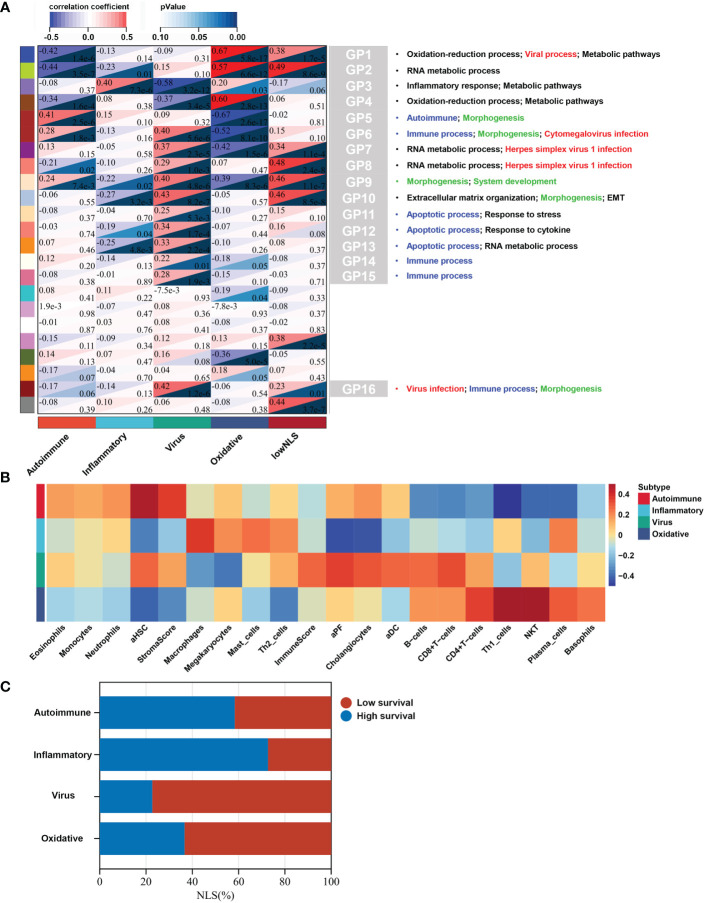
Biological function, biliary fibrosis, immune and prognostic information for each molecular subtype in the training dataset. WGCNA analysis of the training dataset identified gene modules representing different biological processes **(A)**. Correlation heatmap revealed the enrichment scores of immune or biliary fibrotic cells were upregulated in each subtype **(B)**. The percentage of low or high NLS was significant different between these four subtypes **(C)**. aDC, activated dendritic cell; aHSC, activated hepatic stellate cell; aPF, activated portal fibroblast; NLS, natural liver survival; NKT, natural killer T cell; WGCNA, Weighted Gene Co-expression Network Analysis.

To identify biological signatures of individual subtypes, we performed WGCNA and functional enrichment analyses on the training set. The results of WGCNA identified 23 gene modules representing different biological processes (**Figure 3A**). Among these, 16 gene modules were statistically correlated with the four molecular subtypes, and each subtype could be distinguished by these modules. Seven gene modules were associated with NLS. To clarify the biological function of each gene module, we performed functional enrichment analysis of the core genes in the relevant gene module of each subtype. According to the results of the functional analysis, four molecular subtypes were defined: autoimmune, inflammatory, virus infection-related, and oxidative stress. The percentage of the low or high NLS group was also used to assess the results of subtype, and there was a significant difference between these four subtypes, which indicated heterogeneous pathological processes among different subtypes. (**Figure 3C**).

### Autoimmune subtype

**Figure 4:**
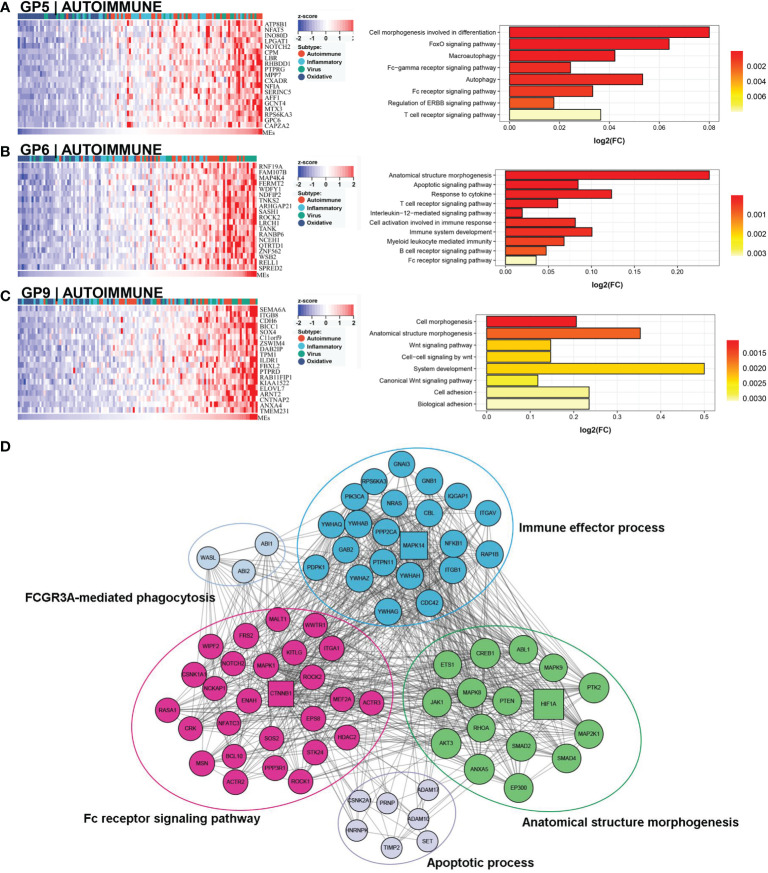
Core GP defining the autoimmune subtype. GP5 revealed autoimmune-related biological processes **(A)**. GP6 revealed immune-related processes **(B)**. GP9 showed biological processes of anatomical morphogenesis **(C)**. The protein-protein interaction network revealed the protein interactions and enriched pathways of representative genes in the autoimmune subtype **(D)**.

This subtype comprised approximately 30% (36/121) of the samples. Patients in this subtype showed a moderate prognosis, with 41.7% (15/36) in the low NLS group (**Figure 3C**). Three gene modules were associated with this subtype (**Figures 4A–C**). Two of the three gene modules were related to immune processes and associated gene programs, including immune system processes, cytokine signaling in the immune system, autophagy, T cell receptor, and apoptotic signaling pathways. And, all three modules showed correlations with biological processes of morphogenesis and tissue development, such as Wnt signaling pathway, anatomical structure and cell morphogenesis. Notably, the Fc-gamma receptor and FOXO signaling pathway, which are involved in the autoimmune process, were significantly enriched in this subtype, which was defined as the autoimmune subtype. The PPI network also revealed the representative molecules of this subtype, such as CTNNB1, HIF1A, MAPK14, and CREB1, which were mainly enriched in immune effector process, FC receptor signaling pathway, and anatomical structure morphogenesis (**Figure 4D**). Moreover, several immune cells associated with autoimmunity, such as eosinophils, monocytes, and neutrophils, were up-regulated in this subtype (**Figure 3B**).

### Inflammatory subtype

**Figure 5:**
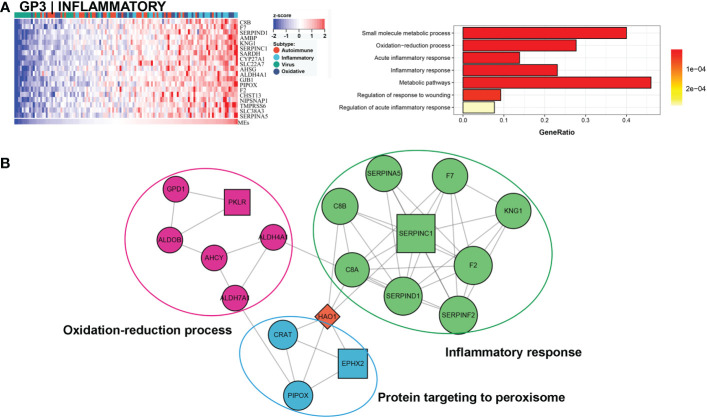
Core GP defining the inflammatory subtype. The main biological processes of GP3 were inflammatory response and metabolic pathways **(A)**. The protein-protein interaction network of hub genes of inflammatory subtype **(B)**.

Approximately 27% (33/121) of BA samples were clustered within this group, which revealed a good prognosis, with 27% (9/33) belonging to the low NLS group (**Figure 3C**). Only one gene module was associated with this subtype, and this gene module was not associated with low NLS (**Figure 3A**). The main biological processes involved were inflammatory responses and metabolic pathways (**Figure 5A**). The representative molecules of this subtype, such as SERPINC1, SERPINF2, PKLR, EPHX2, were also enriched in inflammatory response and oxidation-reduction process (**Figure 5B**). Furthermore, the immune enrichment score for this subtype mainly included macrophages, mast cells, and Th2 cells (**Figure 3B**). Thus, we defined this subtype as inflammatory subtype.

### Virus infection-related subtype

**Figure 6:**
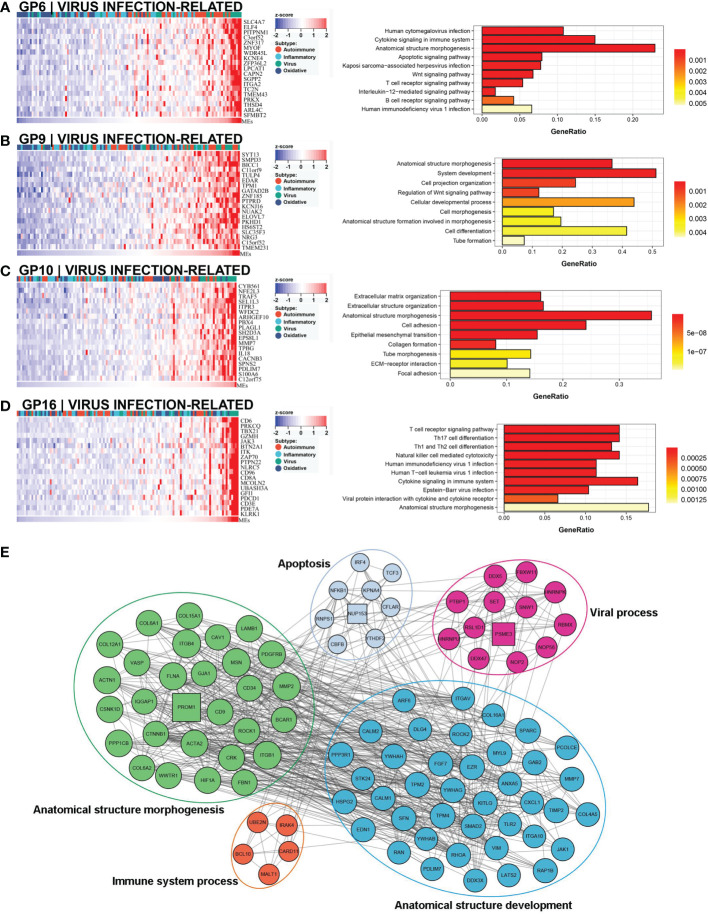
Core GP defining the virus infection-related subtype. The biological processes of GP6 revealed cytomegalovirus infection and immune process **(A)**. GP9 revealed biological processes of anatomical morphogenesis **(B)**. GP10 showed fibrosis-related processes of EMT and ECM **(C)**. The main biological processes of GP16 were virus infection and immune process **(D)**. The protein-protein interaction network of representative genes of virus infection-related subtype revealed the enriched biological processes **(E)**. EMT, epithelial-mesenchymal transition; ECM, extracellular matrix.

A comprehensive functional feature was identified in the third subtype. Approximately 18% (22/121) of the samples were assigned to this subtype with poor prognosis, and 77.3% (17/22) had low NLS (**Figure 3C**). Eleven gene modules were associated with this subtype, and nine of the gene modules were only associated with the subtype (**Figure 3A**). Notably, five of these gene modules were associated with a low NLS. Four gene modules suggested biological processes related to viral infection, including viral protein interaction with cytokines and cytokine receptors, human cytomegalovirus (CMV), and other viral infections (**Figure 6A, D**, and **Supplementary Figures 3A, B**). Additionally, seven gene modules revealed immune-related biological processes such as apoptosis, T cell and B cell activation, and related immune responses (**Figure 6A, D** and **Supplementary Figures 3C–G**). The enriched processes of four gene modules were associated with morphological abnormalities, including anatomical structure morphogenesis, tube morphogenesis, and system development (**Figures 6A–D**). Meanwhile, other gene modules also showed fibrosis-related pathological processes, such as ECM-receptor interaction and epithelial-mesenchymal transition (EMT) (**Figure 6C**). The representative molecules of this subtype such as PROM1, PTBP1, PSME3, NUP153, mainly associated with biological processes including: viral process, immune process, apoptosis, and anatomical structure morphogenesis (**Figure 6E**). The enrichment scores associated with biliary fibrosis, including aHSC, aPF, and cholangiocytes, were significantly increased, andthe immune enrichment score of this subtype also included activated dendritic cell (aDC), B cells, CD4+T cells, and CD8+ T cells (**Figure 3B**). Based on the above biological processes and poor prognosis, we defined this subtype as a virus infection-related subtype.

### Oxidative stress subtype

**Figure 7:**
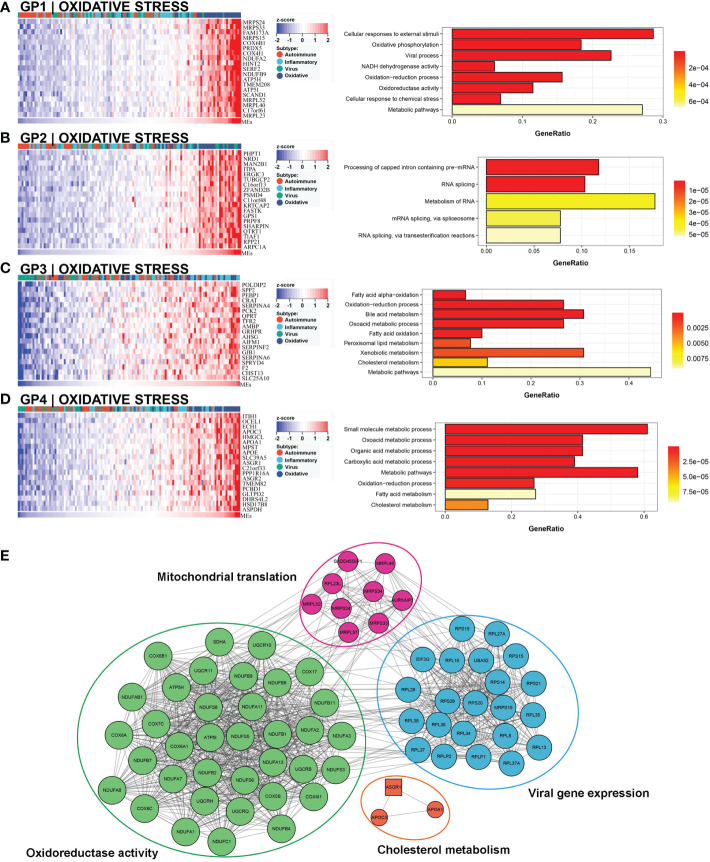
Core GP defining the oxidative stress subtype. GP1 mainly revealed oxidative processes **(A)**. The main biological processes of GP2 were metabolism of RNA **(B)**. The biological processes of GP3 revealed oxidation and metabolism **(C)**. GP4 showed oxidative and metabolic processes **(D)**. The protein-protein interaction network revealed the protein interactions of representative genes in the oxidative subtype **(E)**.

This subtype involved almost 25% (30/121) of the samples, and 63.3% (19/30) of this subtype belonged to the low NLS group (**Figure 3C**). Four gene modules were found to be associated with this subtype. Three of these gene modules were involved in biological processes related to oxidative stress and metabolic processes, including oxidation-reduction processes, oxidative phosphorylation, metabolic pathways, and bile acid metabolism (**Figures 7A–D**). Moreover, one gene module also showed virus-related pathological processes (**Figure 7A**). Meanwhile, the enrichment score for this subtype of natural killer T cells (NKT), basophils, and Th1 cells was significantly increased (**Figure 3B**). The hub molecules represented by NDUFB2, COX5B, and RPS20 were also enriched in similar biological pathways (**Figure 7E**). Therefore, we defined this as the oxidative stress subtype.

### Clinicopathologic characteristics of BA subtypes

**Figure 8:**
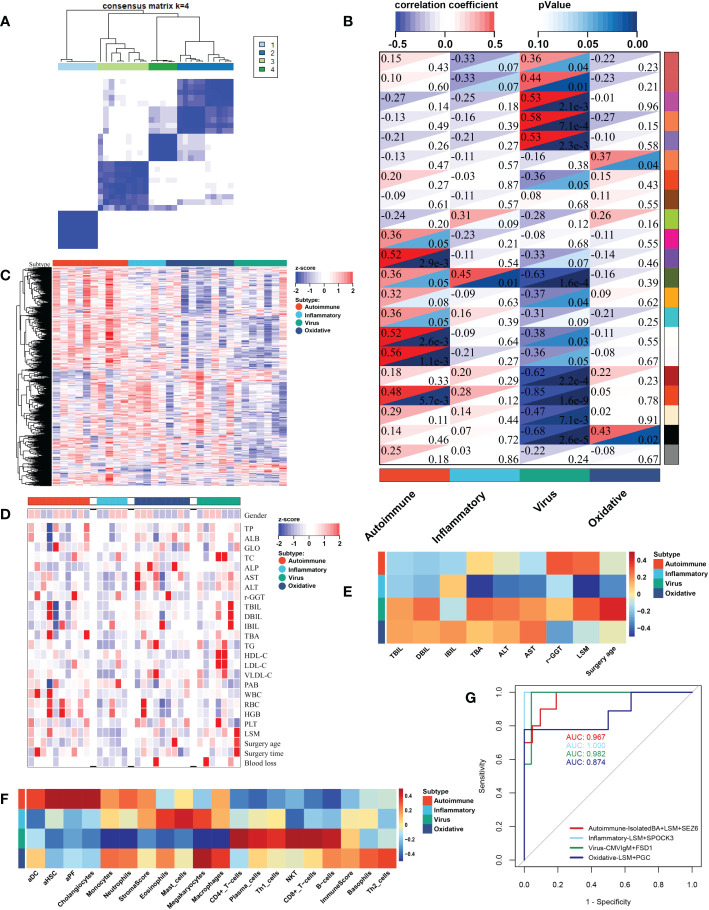
Biological function, fibrosis, immune and clinicopathological information of individual molecular subtypes in our central dataset. By applying SNF-CC, 31 BA patients undergoing HPE in our center were clustered into four distinct subtypes **(A)**. Each subtype was involved with different gene modules of WGCNA **(B)**. GSVA classified the patients into four subtypes **(C)**. Heatmap revealed the clinicopathological features between four molecular subtypes **(D, E)**. The correlation heatmap showed the fibrosis and immune enrichment scores in each subtype **(F)**. The ROC results showed integrated classifier for distinguishing the four molecular subtypes **(G)**. aDC, activated dendritic cell; aHSC, activated hepatic stellate cell; aPF, activated portal fibroblast; ALB, albumin; ALP, alkaline phosphatase; ALT, alanine transaminase; AST, aspartate transaminase; DBIL, direct bilirubin; GLO, globulin; HDL-C, high-density lipoprotein cholesterol; HGB, haemoglobin; IBIL, indirect bilirubin; LDL-C, low-density lipoprotein cholesterol; LSM, liver stiffness measurement; NKT, natural killer T cell; PAB, prealbumin; PLT, platelet; RBC, red blood cell; r-GGT, gamma glutamyl transpeptidase; TBA, total biliary acid; TBIL, total bilirubin; TC, total cholesterol; TG, triglyceride; TP, total protein; VLDL-C, very low-density-lipoprotein cholesterol; WBC, white blood cell; SNF-CC, Similarity Network Fusion and Consensus clustering; GSVA, Gene set variation analysis; WGCNA, Weighted Gene Co-expression Network Analysis; HPE, Hepatoportoenterostomy.

RNA-seq data and clinicopathological information of 31 patients with BA undergoing HPE in our center were used to further validate the reliability and reproducibility of the molecular classification. Similarly, integration analysis of SNF-CC revealed four distinct subtypes with a reliable silhouette width value of 0.83 (**Figure 8A** and **Supplementary Figure 4A**). Likewise, GSVA and WGCNA were applied to detect pathway activity changes within the dataset, which classified the samples into autoimmune, inflammatory, viral infection-related, and oxidative stress (**Figures 8B, C**). The immune cell enrichment scores for each subtype also showed results similar to those of the above datasets (**Figure 8F** and **Supplementary Figure 4B**). Next, we analyzed the clinicopathological features of these subtypes (**Figures 8D, E**, and **Table 1**). As shown in **Table 1**, the difference of LSM and CMV-IgM between these subtypes was statistically significant. Patients with the inflammatory subtype had a lower LSM than those with the other three subtypes. CMV-IgM positive was higher in viral infection-related subtype than the other three subtypes. Notably, some clinicopathological factors showed a trend toward differences between molecular subtypes, although they did not reach statistical significance. The age at surgery for viral infection-related, oxidative stress, and autoimmune diseases was later than that for inflammation. The proportion of embryonal BA was higher in the autoimmune subtype than in the other three subtypes. Additionally, the clearance of jaundice (COJ) at 6 months of viral infection-related oxidative stress was poorer than that of the autoimmune and inflammatory types, which had a similar trend to the above prognostic results. Collectively, these results indicated that there was a heterogeneous clinicopathological process across subtypes.

**Table 1:** Clinicopathological features between four molecular subtypes in 31 BA patients.

Variables	Autoimmune subtype (N=10)	Inflammatory subtype (N=5)	Virus infection-related subtype (N=7)	Oxidative stress subtype (N=9)	*p* value	Effect size	Power
Gender							
Male	6 (60.0%)	3 (60.0%)	3 (42.9%)	4 (44.4%)	0.844	0.163	0.844
Female	4 (40.0%)	2 (40.0%)	4 (57.1%)	5 (55.6%)			
**Birth weight (g)**	3262.00 ± 494.97	3026.00 ± 673.26	3454.29 ± 500.63	3240.00 ± 478.04	0.582	0.271	0.982
**Surgery weight (g)**	4845.00 ± 939.70	4030.00 ± 690.65	5328.57 ± 986.09	4866.67 ± 1127.22	0.186	0.438	0.998
**Surgery age (day)**	57.3 ± 28.27	40.4 ± 14.01	78.43 ± 26.68	66.33 ± 48.949	0.288	0.475	1.000
**AST (U/L)**	210.19 ± 100.02	142.24 ± 94.23	269.27 ± 106.25	285.93 ± 157.83	0.162	0.454	0.999
**ALT (U/L)**	135.68 ± 71.39	82.12 ± 50.90	191.07 ± 85.91	187.10 ± 138.42	0.185	0.438	0.998
**r-GGT (U/L)**	758.42 ± 608.38	469.84 ± 496.75	505.77 ± 267.23	469.13 ± 564.21	0.591	0.268	0.982
**TBIL (μmol/L)**	150.63 ± 84.83	140.65 ± 28.32	191.26 ± 80.75	165.56 ± 64.79	0.533	0.288	0.985
**DBIL (μmol/L)**	103.77 ± 68.30	87.94 ± 31.89	139.26 ± 55.54	112.04 ± 41.04	0.403	0.335	0.992
**IBIL (μmol/L)**	46.86 ± 24.03	52.71 ± 21.24	51.99 ± 28.73	53.93 ± 22.53	0.927	0.130	0.972
**TBA (μmol/L)**	150.29 ± 106.45	82.41 ± 20.59	142.98 ± 41.06	126.49 ± 44.07	0.342	0.359	0.994
**LSM**	13.05 ± 4.27	6.77 ± 1.53	15.00 ± 6.57	10.63 ± 3.65	0.022*	0.648	1.000
Clinical phenotypes							
Isolated	5 (50.0%)	4 (80.0%)	7 (100.0%)	7 (77.8%)	0.244	0.324	0.621
Embryonal	4 (40.0%)	1 (20.0%)	0 (0.0%)	2 (22.2%)			
Cystic	1 (10.0%)	0 (0.0%)	0 (0.0%)	0 (0.0%)			
CMV-IgM							
Positive	1 (10.0%)	0 (0.0%)	4 (57.1%)	1 (11.1%)	0.032*	0.525	0.636
Negative	9 (90.0%)	5 (100.0%)	3 (42.9%)	8 (88.9%)			
Ishak fibrosis score							
1-2	3(30.0%)	4(80.0%)	2(28.6%)	4(44.4%)	0.620	0.267	0.790
3-4	5(50.0%)	1(20.0%)	4(57.1%)	4(44.4%)			
5-6	2(20.0%)	0(0.0%)	1(14.3%)	1(11.1%)			
Recurrent cholangitis							
Yes	2 (20.0%)	1 (20.0%)	2 (28.6%)	3 (33.3%)	0.943	0.134	0.956
No	8 (80.0%)	4 (80.0%)	5 (71.4%)	6 (66.7%)			
NLS at last follow-up							
**Yes**	7 (70.0%)	4 (80.0%)	4 (57.1%)	5 (55.6%)	0.777	0.192	0.859
**No**	3 (30.0%)	1 (20%)	3 (42.9%)	4 (44.4%)			
COJ at 6 months							
Yes	5 (50.0%)	3 (60.0%)	2 (28.6%)	3 (37.5%)	0.687	0.222	0.822
No	5 (50.0%)	2 (40.0%)	5 (71.4%)	5 (62.5%)			

BA, biliary atresia; AST, aspartate transaminase; ALT, alanine transaminase; r-GGT, gamma glutamyl transpeptidase; TBIL, total bilirubin; DBIL, direct bilirubin; IBIL, indirect bilirubin; TBA, total biliary acid; LSM, liver stiffness measurement; NLS, natural liver survival; COJ, clearance of jaundice.

In order to easily identify different molecular subtypes, we combined the molecular and clinical specificity of the subtypes to construct an integrated classifier. The classifier covers three clinical features, including LSM, CMV-IgM, and clinical phenotype, and four molecules. These four molecules are characteristic of each subtype, including: SEZ6 for autoimmune subtype, SPOCK3 for inflammation, FSD1 for viral infection, and PGC for oxidative stress. The ROC results showed that the classifier had high AUC for different subtypes (**Figure 8G**). This confirms that this classifier combining molecular and clinical properties can effectively distinguish the four molecular subtypes. Meanwhile, a series of marker genes were identified to distinguish the four molecular subtypes based on the training cohort. A series of signature genes were identified, including 6 signature genes for autoimmune subtype, 7 for inflammation, 5 for viral infection, and 5 for oxidative stress (**Supplementary Figures 5A, B**). The ROC results showed that the integrated models of these signature genes had higher AUCs in the three cohorts, which confirmed the characteristic genes can distinguish each molecular subtype (**Supplementary Figures 5C–E**).

## Discussion

BA is characterized by extrahepatic biliary obstruction and progressive hepatic fibrosis, related to multiple potential factors that might interact to produce pathological heterogeneity (5). Traditionally, BA has been divided into embryonic and perinatal BA according to the time of disease onset (23). The embryonic BA is rare, which is associated with other congenital malformations such as polyspleen and visceral translocation. More than 80% are perinatal, which may be caused by perinatal factors such as viral infection or autoimmunity. Subsequently, based on morphological and molecular analyses of BA, cyst-associated and CMV-associated BA were added to the clinical phenotypes of BA (24). Several studies have shown a correlation between different clinical phenotypes and NLS (25, 26). However, without knowledge of the etiology and pathogenesis, the traditional phenotype of BA was not suitable for guiding individualized treatment strategies. Most patients with BA underwent same surgical and adjuvant medical treatments despite the coexistence of different clinical phenotypes. In 2010, Moyer et al. used the supervised method of prediction analysis of microarrays (PAM) to classify 43 of 47 infants with BA into inflammation (N = 17) or fibrosis (N = 26) based on molecular profiling and recognized that it might be related to disease staging at diagnosis (27). Recently, Pang et al. classified BA into three subtypes by unsupervised cluster analysis of microarrays: autoimmune, viral, and embryonic subtypes (28). Nonetheless, this study did not address the clinicopathological features and prognosis of BA, which remains an essential step in evaluating the validity and reliability of molecular classification. In the present study, by applying an integrated analysis involving RNA-seq gene expression, biliary fibrosis, and immune index, we identified four BA molecular subtypes and validated them using two independent RNA-seq datasets. These four distinct molecular subtypes exhibited a variety of distinct biological processes related to BA, including viruses, cytokines, oxidative stress, immunity, metabolism, EMT, inflammation, and morphogenesis. It is worth noting that each subtype had distinct prognostic and clinical features, which also confirmed the reliability of molecular subtyping.

The main enriched processes of the autoimmune subtype were immune processes, and morphogenesis. Among these processes, the Fc-gamma receptor and FOXO pathway were significantly activated and associated with various autoimmune diseases in several studies (29, 30). This result is similar to the findings of Pang et al. (28). Furthermore, the immune enrichment scores of eosinophils, monocytes, and neutrophils were also upregulated, which were involved in various autoimmune processes by several studies (31–33). In addition to biological processes related to autoimmunity, enrichment in pathways such as cell morphogenesis and differentiation were also observed in this subtype. Furthermore, consistent with morphogenesis abnormalities, the majority of embryonic BA belonged to the autoimmune subtype. A recent study based on biliary organoids also found that the lack of cholangiocytic differentiation and morphogenesis in BA may be responsible for triggering the immune inflammatory response (34). These results suggest a correlation between abnormal morphogenesis and autoimmunity. In addition to anti-immunotherapy, promoting bile duct epithelial differentiation and morphogenesis may be a way to improve the prognosis of this subtype.

In the inflammatory subtype, the main processes involved were inflammatory responses and metabolic pathways. Additionally, the enrichment scores of macrophages and Th2 cells were significantly upregulated in this subtype, suggesting that the activation of macrophages mediated by Th2 cells could induce inflammatory responses. Studies have reported that patients with BA with Th2 cell-dominated responses have better prognosis (35). In the present study, the inflammatory subtype also had better NLS and COJ. Similarly, Moyer et al. reported that 17 of 47 infants with BA belonged to the inflammatory subtype, which has a better prognosis (27). Because of the better prognosis and liver status, we speculate that patients in this subtype may benefit from repeated HPE if the initial HPE fails. However, we noticed that although no statistical difference was reached, this subtype had a younger age at surgery than the other subtypes, which was similar to the findings of Moyer et al. (27). These results could not rule out whether the inflammatory subtype was BA early stage.

Functional analysis of viral infection-related subtype has revealed various biological processes related to viral infection, such as multiple viral infections, apoptosis, and immune activation. Viral infection, as the primary underlying factor in the mechanism of biliary atresia, has been validated in multiple animal models (36–38). Several studies have reported worse outcomes in patients with BA and CMV infection (24, 39). Similar results were obtained in the present study, with the virus infection-related subtype having the worst NLS and COJ. Notably, our results suggest that, except for viral infection, abnormal morphogenesis, EMT and immune activation might all contribute to the poor prognosis of this subtype. By culturing biliary organoids, Amarachintha et al. likewise proposed that delayed cholangiocytic development and morphogenesis in BA might make it more susceptible to viral damage (34). Similar results were found in this study, virus infection-related subtype was associated with morphogenesis, suggesting that abnormal morphogenesis may be a potential reason why biliary duct cells are more susceptible to viral infection. Additionally, several studies have reported that EMT in bile duct epithelial cells was a key process of biliary fibrosis in BA (40, 41). Factors such as viral infection, bile duct injury, and inflammatory infiltration can induce EMT in bile duct epithelial cells, which eventually transforms into myofibroblasts, leading to biliary fibrosis in BA (41). In the present study, aHSC, aPF, and cholangiocytes were significantly upregulated in the viral infection-related subtype, which reflect the degree of biliary fibrosis (11). A recent study revealed that EMT may be a survival response of bile duct epithelial cells to resist virus-induced apoptosis; however, this response persisted even after virus clearance and eventually led to biliary obstruction (42). These findings are also consistent with the activation of apoptotic and fibrosis-related processes in virus infection-related subtype, including EMT and ECM-receptor interaction. Moreover, various immune processes including B cell, CD4+ T cell, and CD8+ T cell differentiation and activation were significantly upregulated. A recent single-cell sequencing-based study also reported the role of B and T cells in the pathological process of BA, in which depletion of B cells attenuated virus-induced liver injury (43). Based on the above findings, the pathological process of virus infection subtypes might be: abnormal morphogenetic bile duct epithelial cells were induced by virus infection to undergo EMT, and activated apoptotic and immune processes, resulting in severe biliary fibrosis and poor prognosis. Comprehensive therapy including antiviral, anti- immunotherapy, and promotion of biliary morphogenesis may be a way to improve the prognosis of this subtype in the future.

The biological processes involved in the oxidative stress subtype are mainly oxidative stress and metabolic pathways. Several studies have reported that the activation of antioxidant enzymes in BA is increased, suggesting excessive activation of oxidative stress under the stimulation of cholestasis (44, 45). Studies have also confirmed that in the zebrafish model of BA, oxidative stress is a key mechanism leading to biliary injury and obstruction (46, 47). Luo et al. found that patients with BA and low NLS had higher levels of oxidative stress (11). Although oxidative stress and metabolism are dominant in this subtype, some genes are still enriched in viral process pathways. This suggests that a virus-induced oxidative stress process might exist in addition to oxidative disturbances caused by bile acid stasis. In various virus-related diseases, virus-induced oxidative stress is one of the major pathogenic mechanisms of virus-induced inflammation and tissue damage (48–50). The above demonstrates overactivation of oxidative stress in this subtype, which suggests that the application of antioxidant drugs to balance the oxidative state may be an effective strategy for BA.

The present study has several limitations. First, we found that the inflammatory subtype had the best prognosis; however, this subtype also had the youngest age at surgery. We were unable to offset the effect of age at surgery because of the sample size limitations. Therefore, it could not be determined whether this was an independent molecular subtype or early stage of BA. These results require subsequent validation using larger sample sizes. Moreover, although there was a trend towards differences in clinical data across subtypes, most of the characteristics did not reach statistical significance. Some clinical characteristics reached statistical differences, but power analysis showed that the test power needs to be improved by increasing the sample size. This remains to be verified in future studies with larger sample sizes.

In this study, we identified four molecular subtypes of BA that have distinct prognosis, and biological processes, and validated them using multiple datasets. Classification of BA into different molecular subtypes improves our current understanding of the underlying pathogenesis of BA and provides new insights for future studies. Based on the characteristics of the different subtypes, the application of individualized perioperative and preoperative therapies may be a new strategy for improving the prognosis of BA. Accordingly, the results of this study provide new ideas for the treatment of BA in the future; however, further research is needed to verify this.

## Data availability statement

The original contributions presented in the study are included in the article/**Supplementary Material**, further inquiries can be directed to the corresponding author/s.

## Ethics statement

The research protocol was approved by the Ethics Committee of Beijing Children’s Hospital affiliated with Capital Medical University (2019-k-386), and the parents of each patient signed an informed consent form. Written informed consent to participate in this study was provided by the participants’ legal guardian/next of kin.

## Author contributions

DW, SY and JH designed the researches. SY, YZ and YNZ performed the experiments. DW, SY, YZ and TY contributed to the analysis of the data. KH and YG conceived and directed the project. DW, SY, SL, JL and JZ wrote the manuscript with the assistance and feedback of all the other co-authors. All authors contributed to the article and approved the submitted version.

## Supplementary material

Supplementary Figure 1 In the training dataset, the silhouette plot and immune cell index of each subtype and the results of WGCNA. The silhouette plot showed an average silhouette width of 0.85 in the training dataset of 121 BA liver samples **(A)**. The heatmap revealed the fibrosis and immune scores of 121 BA and 7 normal liver samples **(B)**. The cluster dendrogram **(C)**, soft threshold **(D)**, and module eigenvector cluster plot **(E)** of WGCNA in the training dataset. Abbreviation: BA, biliary atresia; WGCNA, Weighted Gene Co-expression Network Analysis

Supplementary Figure 2 In the validation set, the silhouette plot and immune cell index of each subtype and the results of WGCNA. The silhouette plot showed an average silhouette width of 0.77 in the valid dataset of 50 BA liver samples **(A)**. The heatmap revealed the fibrosis and immune scores of validation dataset **(B)**. The cluster dendrogram **(C)**, soft threshold **(D)**, and module eigenvector cluster plot **(E)** of WGCNA in the validation dataset. Abbreviation: BA, biliary atresia; WGCNA, Weighted Gene Co-expression Network Analysis

Supplementary Figure 3 Core GP defining the virus infection-related subtype. The biological processes of GP7 revealed Herpes simplex virus 1 infection and RNA metabolism **(A)**. GP8 revealed biological processes of Herpes simplex virus 1 infection and RNA metabolism **(B)**. GP11 showed fibrosis-related processes of immune and apoptotic processes **(C)**. The main biological processes of GP12 were virus infection and immune process **(D)**. GP13 mainly revealed response to cytokine and apoptotic process **(E)**. The main biological processes of GP14 were immune response **(F)**. The biological processes of GP15 revealed immune cell activations **(G)**.

Supplementary Figure 4 The silhouette plot and immune cell index of each subtype and the results of WGCNA in our center dataset. The silhouette plot showed an average silhouette width of 0.83 in the cohort of 31 BA liver samples in our center **(A)**. The fibrosis and immune scores in 31 BA and 20 normal liver samples **(B)**. The cluster dendrogram **(C)**, soft threshold **(D)**, and module eigenvector cluster plot **(E)** of WGCNA in 31 BA liver samples of our center. Abbreviation: BA, biliary atresia; WGCNA, Weighted Gene Co-expression Network Analysis.

Supplementary Figure 5 Identification of signature genes for BA molecular subtypes. Signature genes for each molecular subtype **(A)**. The expression of signature genes in the training cohort **(B)**. The ROC results of signature gene line model in the training dataset **(C)**. The ROC results of signature gene line model in the validation dataset **(D)**. The ROC results of signature gene line model in our center dataset **(E)**.
