# Supplementary figures and images for: Identifying and validating molecular subtypes of biliary atresia using multiple high-throughput data integration analysis
Source: Front Immunol. 2023 Jan 12;13:1008246. doi: 10.3389/fimmu.2022.1008246 (PMC9878701; doi:10.3389/fimmu.2022.1008246)

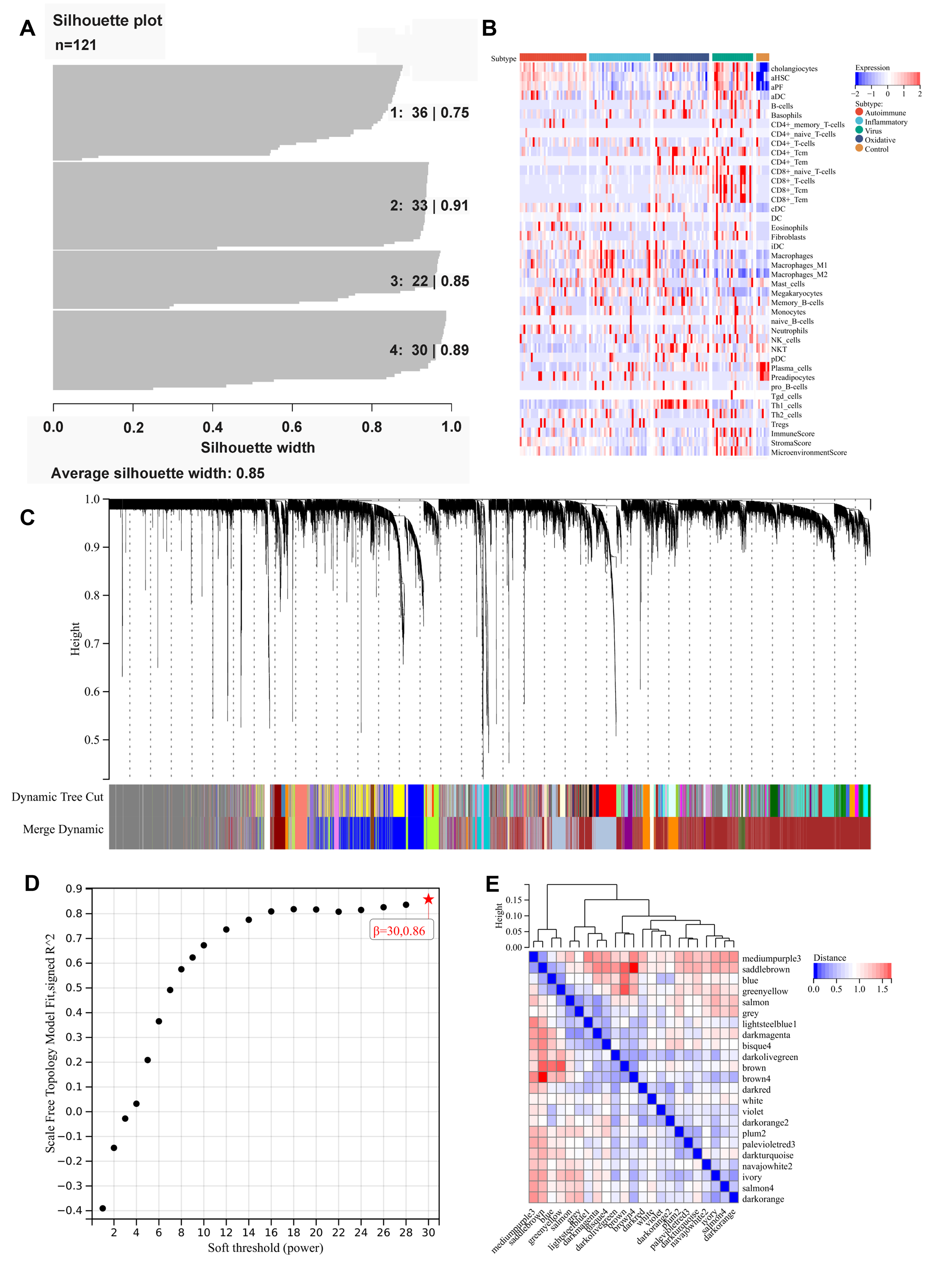

Supplement: Supplementary Figure 1 — In the training dataset, the silhouette plot and immune cell index of each subtype and the results of WGCNA. The silhouette plot showed an average silhouette width of 0.85 in the training dataset of 121 BA liver samples (A). The heatmap revealed the fibrosis and immune scores of 121 BA and 7 normal liver samples (B). The cluster dendrogram (C), soft threshold (D), and module eigenvector cluster plot (E) of WGCNA in the training dataset. Abbreviation: BA, biliary atresia; WGCNA, Weighted Gene Co-expression Network Analysis [file Image_1.tif]

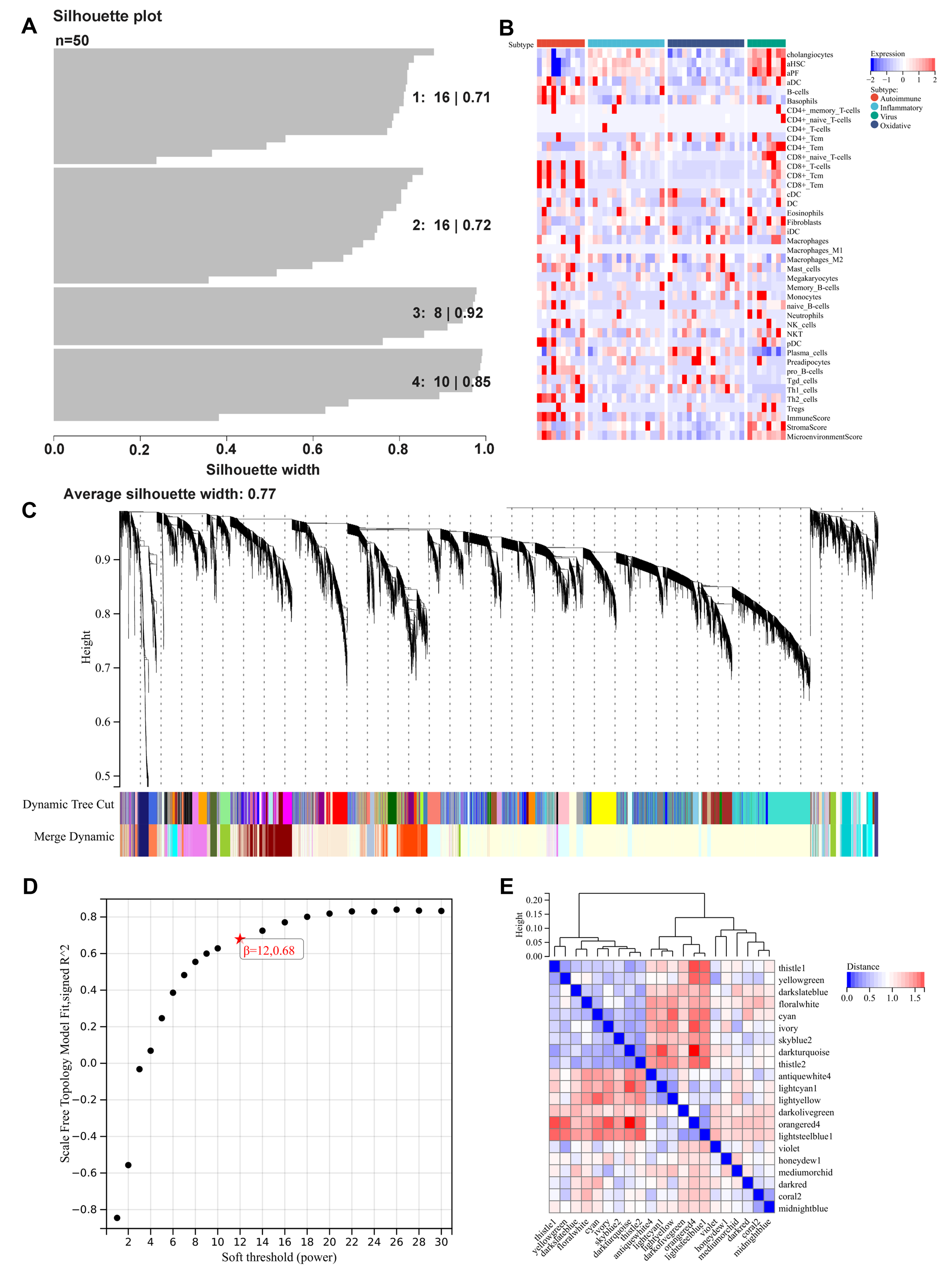

Supplement: Supplementary Figure 2 — In the validation set, the silhouette plot and immune cell index of each subtype and the results of WGCNA. The silhouette plot showed an average silhouette width of 0.77 in the valid dataset of 50 BA liver samples (A). The heatmap revealed the fibrosis and immune scores of validation dataset (B). The cluster dendrogram (C), soft threshold (D), and module eigenvector cluster plot (E) of WGCNA in the validation dataset. Abbreviation: BA, biliary atresia; WGCNA, Weighted Gene Co-expression Network Analysis [file Image_2.tif]

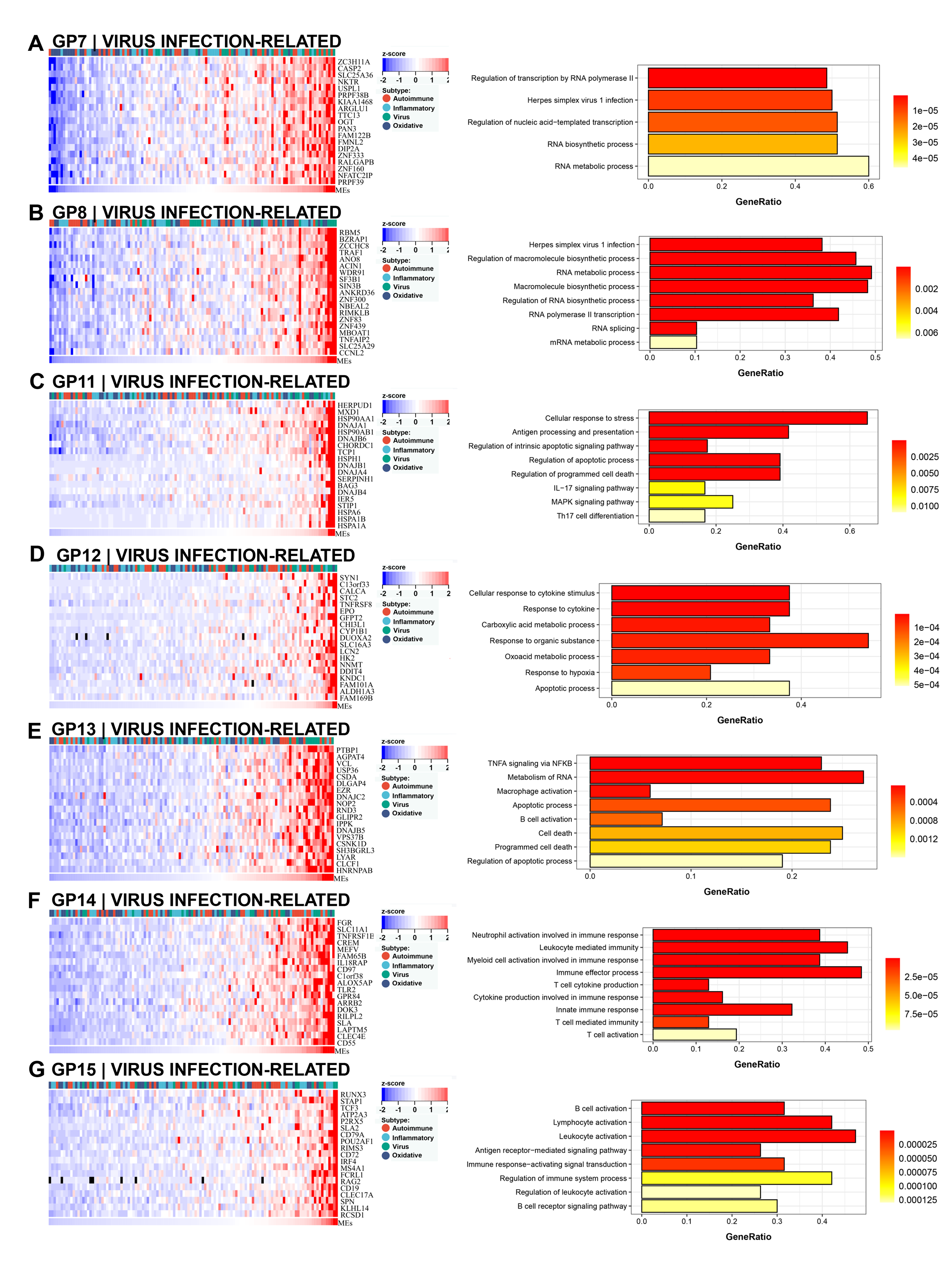

Supplement: Supplementary Figure 3 — Core GP defining the virus infection-related subtype. The biological processes of GP7 revealed Herpes simplex virus 1 infection and RNA metabolism (A). GP8 revealed biological processes of Herpes simplex virus 1 infection and RNA metabolism (B). GP11 showed fibrosis-related processes of immune and apoptotic processes (C). The main biological processes of GP12 were virus infection and immune process (D). GP13 mainly revealed response to cytokine and apoptotic process (E). The main biological processes of GP14 were immune response (F). The biological processes of GP15 revealed immune cell activations (G). [file Image_3.tif]

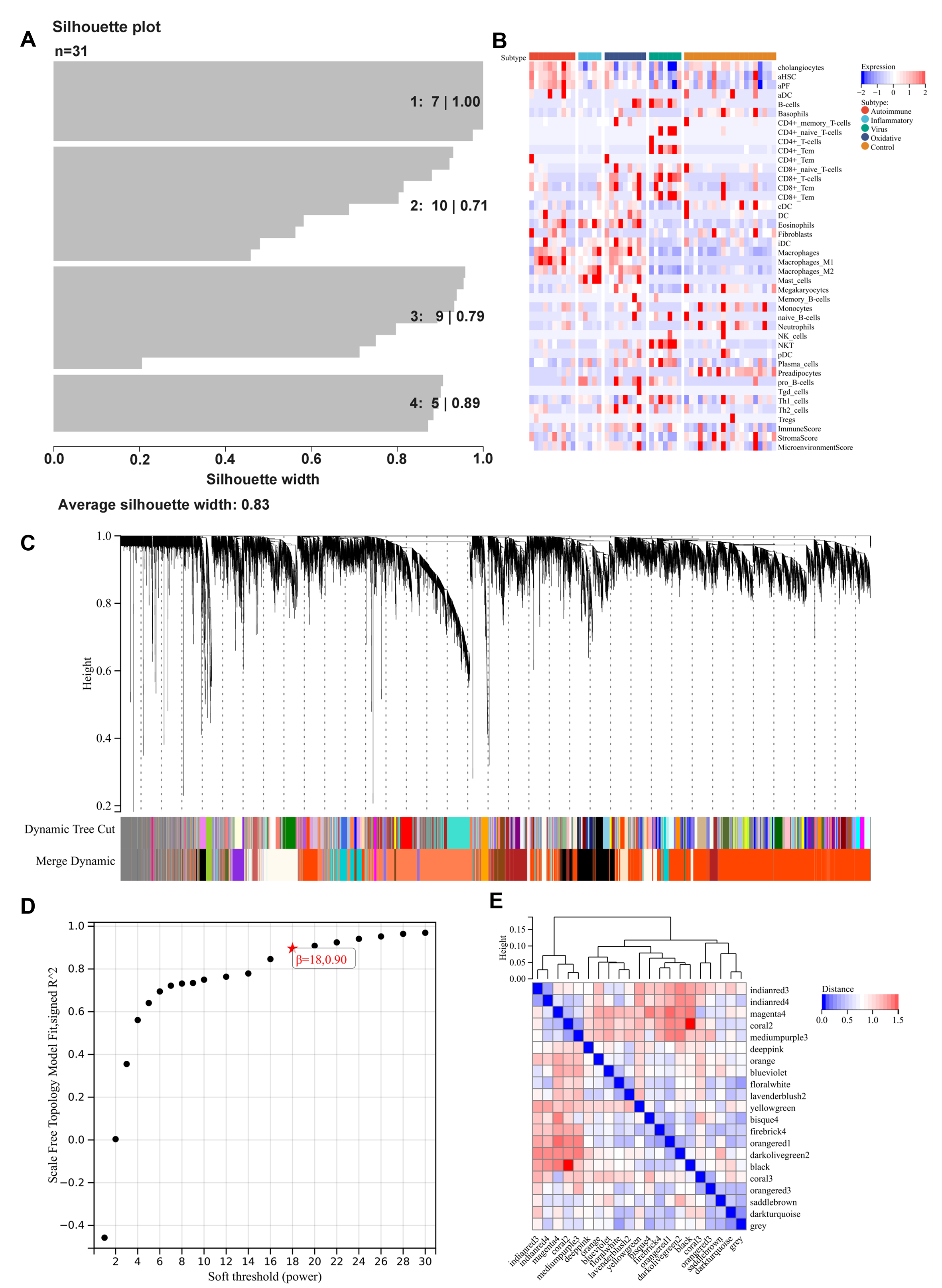

Supplement: Supplementary Figure 4 — The silhouette plot and immune cell index of each subtype and the results of WGCNA in our center dataset. The silhouette plot showed an average silhouette width of 0.83 in the cohort of 31 BA liver samples in our center (A). The fibrosis and immune scores in 31 BA and 20 normal liver samples (B). The cluster dendrogram (C), soft threshold (D), and module eigenvector cluster plot (E) of WGCNA in 31 BA liver samples of our center. Abbreviation: BA, biliary atresia; WGCNA, Weighted Gene Co-expression Network Analysis. [file Image_4.tif]

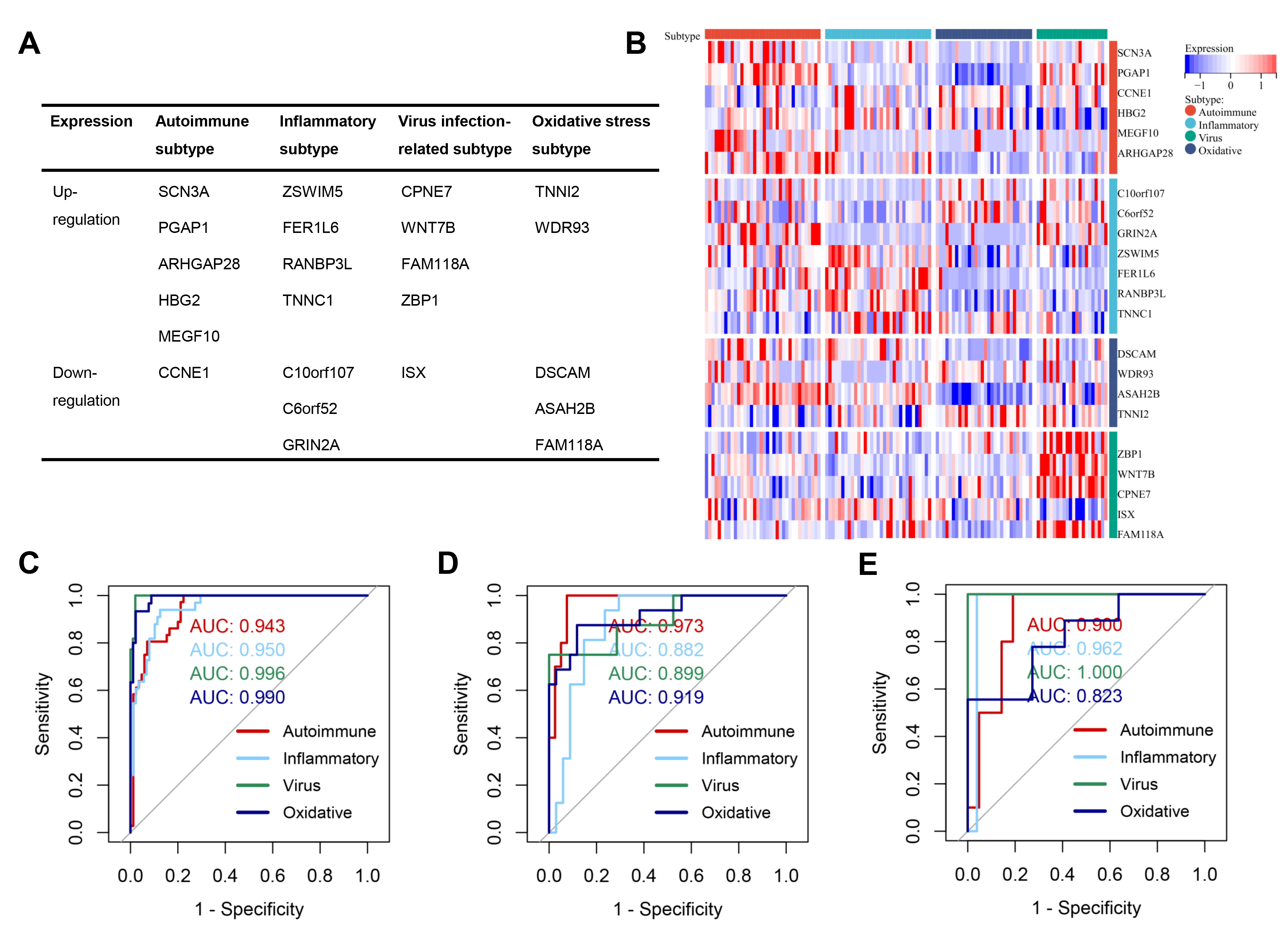

Supplement: Supplementary Figure 5 — Identification of signature genes for BA molecular subtypes. Signature genes for each molecular subtype (A). The expression of signature genes in the training cohort (B). The ROC results of signature gene line model in the training dataset (C). The ROC results of signature gene line model in the validation dataset (D). The ROC results of signature gene line model in our center dataset (E). [file Image_5.tif]
